# Insights into the Cyanobacterial Deg/HtrA Proteases

**DOI:** 10.3389/fpls.2016.00694

**Published:** 2016-05-24

**Authors:** Otilia Cheregi, Raik Wagner, Christiane Funk

**Affiliations:** Department of Chemistry, Umeå UniversityUmeå, Sweden

**Keywords:** cyanobacteria, serine proteases, phylogeny, PSII degradation, chaperone, secretion, cell surface

## Abstract

Proteins are the main machinery for all living processes in a cell; they provide structural elements, regulate biochemical reactions as enzymes, and are the interface to the outside as receptors and transporters. Like any other machinery proteins have to be assembled correctly and need maintenance after damage, e.g., caused by changes in environmental conditions, genetic mutations, and limitations in the availability of cofactors. Proteases and chaperones help in repair, assembly, and folding of damaged and misfolded protein complexes cost-effective, with low energy investment compared with neo-synthesis. Despite their importance for viability, the specific biological role of most proteases *in vivo* is largely unknown. Deg/HtrA proteases, a family of serine-type ATP-independent proteases, have been shown in higher plants to be involved in the degradation of the Photosystem II reaction center protein D1. The objective of this review is to highlight the structure and function of their cyanobacterial orthologs. Homology modeling was used to find specific features of the SynDeg/HtrA proteases of *Synechocystis* sp. PCC 6803. Based on the available data concerning their location and their physiological substrates we conclude that these Deg proteases not only have important housekeeping and chaperone functions within the cell, but also are needed for remodeling the cell exterior.

## Introduction

Proteolysis, associated with nutrient uptake, processing and activation of proteins, or removal of damaged proteins, is vital for every cell. Cells therefore have developed a sophisticated system of molecular chaperones and proteases to reduce the amount of unfolded or aggregated proteins (Wickner et al., 1999). Proteases belonging to the Deg (for *deg*radation of periplasmic proteins) or HtrA (for *h*igh *t*emperature *r*equirement *A*) family are known in all kingdoms, in Bacteria, Eukarya, and even some Archaea (Koonin and Aravind, 2002; Page and Di Cera, 2008). These serine proteases belong, according to the MEROPS nomenclature, to the S1C subfamily of the clan PA (Rawlings et al., 2012), consisting of endopeptidases with the catalytic residues histidine, aspartate, and with serine or cysteine as nucleophile. Families are assigned to the S1 subfamily when they are similar to chymotrypsin; S1C contains Deg peptidases and their homologs. Degs are ATP-independent serine endopeptidases, which besides the trypsin-type protease domain possess 0–4 PDZ or PDZ-like domains toward their C-terminus (Pallen and Wren, 1997; Clausen et al., 2002). PDZ domains mediate protein–protein interactions and are important for substrate recognition and/or for the regulation of proteolytic activity (Krojer et al., 2002; Wilken et al., 2004), the PDZ domains of Deg/HtrA proteases even have been shown to act as enzymatic co-factors (see Clausen et al., 2011). While commonly serine proteases consist of monomers, members of the Deg/HtrA family form homotrimers. One distinctive property of Deg/HtrAs is their ability to self-compartmentize and assemble into complex oligomers. The assembly and disassembly of higher-order oligomers allows for precise regulation of protease activity.

Eukaryotic Deg proteases take care of damaged cellular components, act as tumor repressors, or protect the cell from the accumulation of toxic protein aggregates responsible for neurodegeneration in, e.g., Alzheimer’s and Parkinson’s diseases. Most prokaryotic Deg/HtrA proteases play critical roles in protein quality control within the periplasmic compartment. Several members of the family even have explicit functions in bacterial pathogenicity and are involved in biofilm formation (Biswas and Biswas, 2005), secretion of virulence factors (Baud et al., 2009) and/or remodeling of cell–cell contacts within the host organisms (Hoy et al., 2010). *Escherichia coli* possesses three Deg/HtrA proteases, EcDegP (or EcHtrA), EcDegQ (or EcHhoA) and DegS (or EcHhoB). EcDegP seems to be responsible for maintaining the periplasmic protein homeostasis and thus is crucial for survival under stress conditions such as higher temperatures (Lipinska et al., 1988); EcDegS cleaves the transmembrane RseA protein in the cytoplasmic membrane, which binds and inhibits the σ^E^ factor (Walsh et al., 2003). EcDegQ of *E. coli* is a periplasmic protease with similar functions to EcDegP (Pallen and Wren, 1997). Contrary to *E. coli* many prokaryotes encode only a DegQ homolog, stressing the importance of this protease (Kim and Kim, 2005).

Less information is available on Deg proteases from photosynthetic organisms. In the genome of *Arabidopsis thaliana*, 16 *DEG* protease genes have been identified (reviewed in Huesgen et al., 2009; Schuhmann et al., 2012), 15 in *Oryza sativa* (Tripathi and Sowdhamini, 2006), and 20 in *Populus trichocarpa* (Garcia-Lorenzo et al., 2006). In *A. thaliana* (hereafter *Arabidopsis*) at least five AtDeg proteases (AtDeg1, AtDeg2, AtDeg5, AtDeg7, and AtDeg8) are located in the chloroplast (Schuhmann et al., 2012; Tanz et al., 2014). Of those, the Deg proteases located in the thylakoid lumen (AtDeg1, AtDeg5, and AtDeg8), as well as the mitochondrion-located AtDeg14 (Tanz et al., 2014), share high homology to cyanobacterial Deg proteases (Schuhmann et al., 2012).

For detailed information on human, bacterial and plant Deg proteases the reader is referred to the excellent review by Clausen et al. (2011). In this review, we will focus on cyanobacterial Deg proteases, which generally have been neglected in other reviews. Special focus will be on the three Deg proteases SynHhoA, SynHhoB, and SynHtrA of *Synechocystis sp.* PCC 6803 (hereafter *Synechocystis* 6803). These SynDeg proteases relate more to each other than to their homologs in *E. coli* (see Kieselbach and Funk, 2003), making it impossible to assign functions based on homology studies. They are encoded by *sll1679* (SynHhoA), *sll1427* (SynHhoB), and *slr1204* (SynHtrA), but their function within the cyanobacterial cell still is not known. Even the subcellular location of the three cyanobacterial SynDeg proteases remains enigmatic. In the following sections, we will review the current knowledge of the *Synechocystis* 6803 SynDeg proteases and compare these data with Deg proteases of other organisms.

## The Family of Deg Proteases in Cyanobacteria – A Phylogenetic Comparison

Within cyanobacteria biochemical or molecular biological information is available only on the SynDeg proteases of *Synechocystis* 6803. The cyanobacterial phylogeny has been recently re-determined based on new data obtained using modern techniques, e.g., comparative mass genomics, comparison of conserved proteins and resulting secondary metabolites; more than 200 cyanobacterial genomes have been sequenced (Micallef et al., 2015). A subset of those, stretching over the whole phylum, was here investigated in the search for Deg proteases (InterPro domain IPR001940^1^). The majority of the cyanobacterial strains contain three to five genes encoding members of the Deg family, however, up to seven genes are present in, e.g., *Cyanothece sp*. PCC 7425 or *Nostoc punctiforme* ATCC 29133, while several *Prochlorococcus* strains only contain two genes. Based on the analyzed species it seems that cyanobacterial Deg proteases only contain one PDZ domain.

**FIGURE 1:**
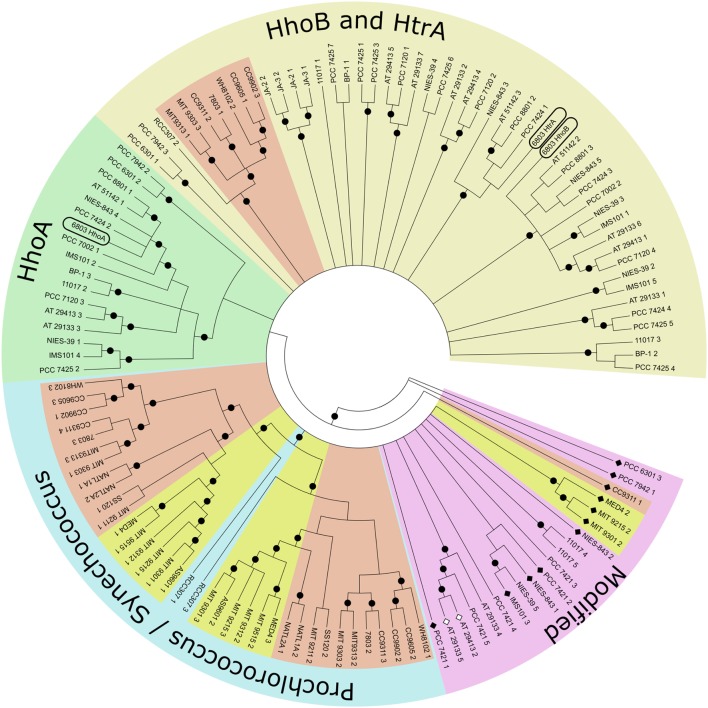
**Topological bootstrap consensus tree of cyanobacterial Deg proteases.** Phylogenetic tree built using the Maximum Likelihood method from predicted mature protein sequences. The bootstrap consensus tree was inferred from 1000 replicates. Full taxonomic names can be found in **Supplementary Table S1**. The last number of each taxon indicates the gene copy of the Deg peptidase of that species. Light yellow: cluster containing *Synechocystis* 6803 HhoB and HtrA. Light green section contains HhoA of *Synechocystis* 6803. Light blue circle section contains species of the evolutionary distinct monophyletic groups *Prochlorococcus* and *Synechococcus*. Species of *Prochlorococcus* are known to be high light (yellow) or low light (red) adapted. Also low light adapted *Synechococcus* are marked in red. In the light purple circle section cluster proteins that are (a) very ancient like *Gloeobacter* sequences, (b) Deg peptidases without a PDZ domain (black diamonds), (c) inactive Deg peptidases (white diamonds, ATCC 29133, ATCC 29413), or (d) peptidases with additional elements for unknown reasons. Black circles indicate branches with a frequency of 70% and more.

Using the maximum likelihood method to generate a bootstrap consensus tree it becomes obvious that Deg proteases do not cluster in their phylogenetic subclades (**Figure 1**, name abbreviations are explained in **Supplementary Table S1**; Calteau et al., 2014). Looking at a wide variety of species, orthologs to SynHhoB and SynHtrA of *Synechocystis* 6803 form one cluster, while SynHhoA-homologs form a distinct, different one. Notably, genes encoding Deg peptidases in the *Prochlorococcus* and *Synechococcus* branch highlight the evolutionary development within this group. *Deg* genes of *Prochlorococcus* have common ancestors with the low-light adapted *Synechococcus* strains, i.e., *Synechococcus* sp. RCC307. RCC307 and several early species contain three genes encoding Deg proteases: one of a HhoB/HtrA type and two distinct ones, forming the base of the *Prochlorococcus/Synechococcus* clade. *Synechococcus* sp. CC9311 contains additionally a gene coding for a modified Deg lacking its PDZ domain. Its protease domain is similar to the “specialized” Degs of the *Synechococcus* branch. Some low-light adapted *Prochlorococcus* strains like *Prochlorococcus marinus* SS120 or NATL1A have lost their HhoB/HtrA type peptidase. Even the high-light adapted strains underwent massive genetic restructuring (Kettler et al., 2007), which affected the Deg peptidases. Strains like *Prochlorococcus marinus* MED4, MIT9215, and MIT9301 lost the PDZ domain of their HhoB/HtrA type Deg, while this HhoB/HtrA type is lacking completely in MIT9312, MIT9215, and AS9601. Genes encoding Deg peptidases of *Prochlorococcus marinus* lacking a PDZ domain cluster with “unusual” *deg* genes of very ancient species like *Gloeobacter* but also evolutionary young *Nostocales*. Even *deg* genes with additional unknown, or mutated peptidase domains, rendering the gene product proteolytically inactive (*Nostoc punctiforme* ATCC 29133, *Anabaena variabilis* ATCC 29413) are within this cluster. These enzymes might have undergone neo-functionalization.

## Structural Implications of Degs from Photosynthetic Organisms

Of all photosynthetic organisms only the structures of recombinant *Arabidopsis* AtDeg1, AtDeg2, AtDeg5, and AtDeg8 have been published. The domain architectures of all Deg proteases are well conserved (Kieselbach and Funk, 2003; Page and Di Cera, 2008). Most family members contain either an amino terminal transit peptide or a transmembrane-spanning helix followed by the structurally conserved peptidase domain (see **Supplementary Table S1**). This peptidase domain consists of two β-barrels perpendicular to each other, build by twelve β-strands connected by loops. Usually two longer α-helices enclose the barrel structure, additional smaller helices and β-strands can be found in the available crystal structures of Deg peptidases. The amino acids of the catalytic triad, His, Asp and Ser, are placed within the cleft between the β-barrels. Three of the five loops of the peptidase domain are needed to form the active site: the LD loop is the activation loop, L1 contains the serine residue of the catalytic triad and forms the oxyanion hole, while L2 forms a substrate specific pocket. Loops L3 and LA are regulatory loops for activation via oligomerization (Clausen et al., 2011). L3 is called sensor loop, it is important to initiate the activation cascade. The LA loop is involved in hexamer formation of the soluble Deg peptidases. Additionally regulatory elements can be found; AtDeg1 of *Arabidopsis*, for example, sense the pH via a special histidine residue in its peptidase domain, while human HTRA1, HTRA3, and HTRA4 contain fragments of an insulin growth factor binding protein (Clausen et al., 2002).

**FIGURE 2:**
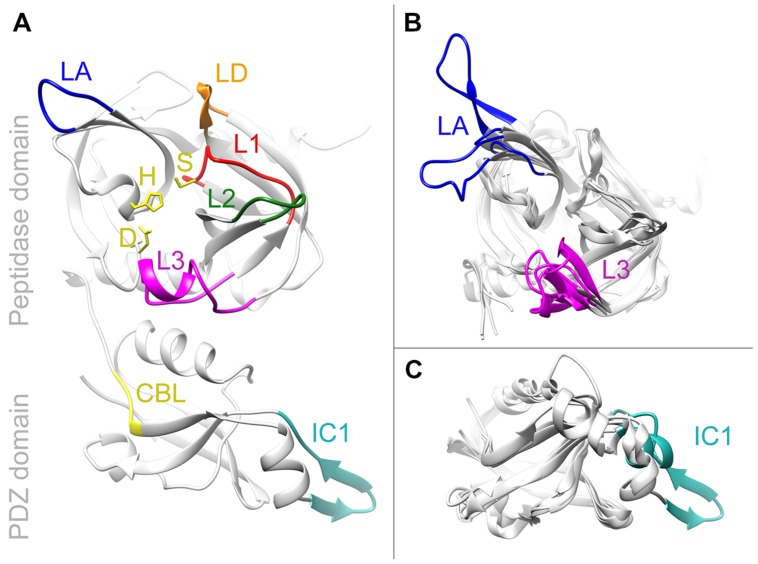
**Homology models of HhoB.** Models were obtained by Phyre2. **(A)** SynHhoB modeled after 3STJ, the crystal structure derived of *E. coli* DegQ. Side chains of the catalytic triad (H151, D181, and S258) are shown in yellow. They are surrounded by loops forming the active site: activation loop LD in orange, loop for the oxyanion hole L1 in red, and the substrate specific loop L2 in green. The regulator loops are LA (blue) and L3 (magenta). Of the PDZ domain the carboxylate binding loop (CBL) is given in yellow and the interaction clamp one (IC1) in cyan. **(B,C)** Superposition of peptidase domain **(B)** or PDZ domain **(C)** using different HhoB models. These models were formed using information of the crystal structures of DegQ, Deg1, Deg2, and Deg8. Color coding is the same as in **(A)**. Only varying elements were colored.

The Deg proteases of photosynthetic organisms contain up to two PDZ domains, which are connected via a short linker to the C-terminal end of the peptidase domain (Kim and Sheng, 2004). PDZ domains set apart the Deg subfamily from other members of the MEROPS S1 family; they are able to bind to the carboxyl-termini of proteins or they can dimerize with other PDZ domains. Cyanobacterial Degs as well as AtDeg1 and AtDeg8 of *Arabidopsis* contain only one PDZ domain; AtDeg2 has two PDZ domains. Its second PDZ domain (PDZ2) is not able to bind substrate due to a conserved internal ligand occupying the peptide binding site, instead this internal ligand is part of the docking site for the LA loop to form a stable hexamer (Sun et al., 2012). AtDeg15 and AtDeg5 of *Arabidopsis* do not contain any PDZ domains. AtDeg15 acts as glyoxysomal processing peptidase (Helm et al., 2007), while AtDeg5 forms peptidase-substrate complexes together with AtDeg8 (Sun et al., 2007). PDZ domains are compact globular domains made up canonical by six β-sheets and two α-helices (Clausen et al., 2011). The first element of PDZ domains in Deg peptidases is the carboxylate-binding loop, which interacts with the C-termini of substrate proteins and presents those to the proteolytic active site. This loop usually is composed of a GLGF motif, but in cyanobacteria the first position often is replaced by phenylalanine or tyrosine, while positions two and four are either isoleucine or valine. The motif is followed by the peptide-binding site formed of a β-strand and an α-helix. PDZ domains of cyanobacteria contain at least one interaction clamp (IC1) for protein-protein interaction (Clausen et al., 2002; Krojer et al., 2008). Plant Deg1 even contains a second interaction clamp (IC2), four of its residues seem to be involved in interaction (Kley et al., 2011). Sequence alignments highlight that this motif is only found in higher plants. However, other residues might be important for oligomerization of cyanobacterial Deg proteases. IC1s of cyanobacteria seem to contain additional residues resulting in a varying structure after homology modeling (see **Figure 2**).

The basic unit of Deg proteases in their tertiary structure is a homotrimer formed by hydrophobic interactions and hydrogen bonds of the peptidase domains. The trimeric form resembles a meniscus lens or a shallow funnel with the active sites located at the center of the concave site, while the PDZ domains are on the rim of the trimer. Depending on the activation mechanism, the organism, and the functional state Deg peptidases can oligomerize into larger structures via intramolecular PDZ–PDZ interactions (for a review, see Hansen and Hilgenfeld, 2013). Hexamers, nonamers, dodecamers, octodecamers, and 24-mers were detected using size exclusion chromatography or crystallography (Krojer et al., 2008; Huesgen et al., 2011). For AtDeg1 a pH-dependent oligomerization has been observed; inactive monomers oligomerize into trimers at acidic pH. These trimers furthermore assemble into hexamers by interaction of IC1 and IC2 with the closest LA loop of the opposing trimer. By formation of these hexamers the PDZ domains are fixed; the PDZ–L3–LD interaction network establishes a defined opening of the hexameric cage and stabilizes the active site (Kley et al., 2011). Reorientation of LD by L3 of the neighboring protomer allosterically activates the Deg peptidase and is ubiquitous among this subfamily (Clausen et al., 2011). The resting state of AtDeg2 is suggested to be a hexamer, incubation with the substitute-substrate β-casein then leads to the formation of higher oligomeric states. The first PDZ domain (PDZ1) binds the substrate and presents it to the active site, while PDZ2 becomes fixed and forms an LA-docking surface. Interactions of PDZ2 with PDZ1 of the opposing trimer lead to trimer-dimerization. It is suggested that PDZ2 can also unlock the hexameric state to re-assemble into an active dodecamer in the presence of substrate (Sun et al., 2012). AtDeg5, lacking any PDZ domains, forms trimers in solution that bind two calcium ions along the channel between the protomers. These calcium ions are dispensable for trimer formation (Sun et al., 2013). AtDeg8 exists as monomer and trimer at pH 6.0, while hexamers are formed at pH 8.0. The crystal structure of hexameric AtDeg8 shows flattened PDZ domains resulting in a flatter funnel formation and a narrower cavity of the hexamer compared to other structures, substrate access to its active site seems to be prevented (Sun et al., 2013). Furthermore, in the resolved crystal structures of AtDeg5 and AtDeg8 the histidines of the catalytic triad are rotated and the L2 loop seems to be differently formed pointing to a different activation mechanism (Sun et al., 2013). AtDeg5 and AtDeg8 are so far the only Deg proteases known to form hetero-oligomers out of homo-trimers (Sun et al., 2007).

The PDZ domain of the three SynDeg proteases of *Synechocystis* 6803 is needed for oligomerization beyond homotrimers (Huesgen et al., 2011). While recombinant SynHhoB does not form higher oligomers, SynHhoA aggregates into 12–24-mers, and SynHtrA into 9–12 protomers. Interestingly, recombinant SynDegs lacking their PDZ domains were still proteolytically active, implying that in *Synechocystis* 6803 the PDZ domain is not needed as signal for L3 reorientation to form the active site (Huesgen et al., 2011). So far no crystallographic structures of cyanobacterial Deg peptidases have been published. Homology modeling was performed using *Thermotoga maritima* HtrA and *E. coli* EcDegS as template (Jansén et al., 2005); today many more Deg structures of various organisms are known. We used the intense mode of Phyre2 (Kelley et al., 2015) for homology modeling of *Synechocystis* 6803 SynHhoB (Sll1427). Phyre2 returned several models with high confidence, from those SynHhoB modeled after *E. coli* EcDegQ, RSCB-ID 3STJ, chain C (Sawa et al., 2011) was picked as template, because this protein has been crystallized in its trimeric form and *Synechocystis* 6803 SynHhoB forms trimers in solutions (Huesgen et al., 2011), (**Figure 2**). Superposition of SynHhoB modeled after AtDeg1 (3qo6), AtDeg2 (4fln), and AtDeg8 (4ic6) using EcDegQ as template (Kley et al., 2011; Sun et al., 2012, 2013) is shown in **Figures 2B,C**. Due to different positioning of the PDZ domains in the known Deg structures the peptidase domain was independently modeled from the PDZ domain. The basic Deg peptidase elements, perpendicular β-barrels with the active site consisting of the catalytic triad and loops LD, L1, and L2 are conserved in the model of *Synechocystis* 6803 SynHhoB. Sterical variations can be observed in loops LA and L3 of the peptidase domain, as well as in IC1 and the substrate binding helix. In *Synechocystis* SynHhoB LA loop and IC1 motif seem to be longer compared to *Arabidopsis* AtDegs. The Phyre2s method includes prediction and modeling of secondary structures, the variety of IC1 therefore could induce the observed changes in the substrate binding helix at the N-terminus. Differences in the activation state of the template Deg proteases explains the observed variation of the *Synechocystis* 6803 L3 loop. For more detailed structural comparisons of cyanobacterial Deg proteases we have to await the resolution of their crystal structures.

## Regulation of the Deg Proteases

### Transcriptional Regulation

The three *Syn-deg* genes are found in the *Synechocystis* 6803 genome at distinct locations and belong to transcriptional units (TU) including 1–3 genes (Kopf et al., 2014). *SynHhoA* is part of an operon with *sll1680* and *sll1681*, encoding a hypothetical protein and a protein of unknown function. Its mRNA is transcribed with a length of 3800 bp. Antisense RNA (aRNA) overlapping the 3′ end of *Syn-hhoA* is expressed at higher levels than *Syn-hhoA* itself, in all conditions tested (Kopf et al., 2014). mRNA of *Syn-hhoB* has a length of 1510 bp with extra 239 bp at the 3′ end and 21 bp at the 5′ end. Even within *Syn-hhoB* antisense RNA is located on the complementary strand, toward the 3′ end, which is expressed at lower levels than *Syn-hhoB* (Kopf et al., 2014). *Syn-htrA* is part of a TU of 1500 bp, with a short 3′ UTR (47 bp) and a longer 5′ UTR end of 122 bp. The antisense RNA located complementary to this gene, *ncr0370*, is, similar to aRNA of *Syn-hhoB*, overlapping the 3′ end of the gene and its 3′ UTR. *Ncr0370* has maximal reads in the exponential phase, about 10 times higher than *Syn-htrA*. In the nine stress conditions investigated (Kopf et al., 2014), *Syn-htrA* is induced in each experiment about eight times, while the expression of the non-coding RNA declines, pointing to possible functional correlations. Contrary to the strong up-regulation of *Syn-htrA* in all investigated stress conditions (cold, Fe limitation, high light stress, darkness, heat shock, stationary phase, C depletion, N depletion and P depletion, Kopf et al., 2014), the expression of *Syn-hhoA* and *Syn-hhoB* are low. According to the study of Mrázek et al. (2001), which predicts gene expression level from relative codon usage bias, all three *Syn-deg* genes are predicted to be lower expressed than other members of the chaperone/degradation machinery such as the genes encoding DnaK, ClpC, FtsH, and CtpA proteases. Microarray analyses, taking different stress conditions and different genetic backgrounds in consideration, support these results (Hernandez-Prieto and Futschik, 2012; Kopf et al., 2014) (**Supplementary Table S2**). Only few abiotic stresses significantly induce *Syn-deg* genes: 2 h treatment of UV-B light combined with high light intensity (Huang et al., 2002b) and 30 min growth in salt (0.5 M NaCl) or hyperosmotic stress (0.5 M sorbitol; Kanesaki et al., 2002). Stressing *Synechocystis* 6803 cells with high concentrations of Cd^2+^ affects primarily the level of all protease-encoding genes, *Syn-htrA* being among the most strongly induced already after 30 min of stress (Houot et al., 2007). *Syn-htrA* and *Syn-hhoA* genes were found to be induced in a Δ*psbO*:Δ*psbU* pseudorevertant that is able to grow photo-autotrophically at pH ∼7.5 (Summerfield et al., 2007). In this work, *Syn-hhoA* and *Syn-htrA* seem to be part of a stress-response network, which balances the instability of PSII caused by the deletion of *psbO* and *psbV* (Summerfield et al., 2007). Co-expression analysis has demonstrated that *Syn-hhoA*, *Syn-hhoB*, and *Syn-htrA* belong to distinct co-expression clusters suggesting unique physiological functions (Miranda et al., 2013), besides their proposed overlapping functions (Barker et al., 2006). *Syn-htrA* is co-expressed with genes coding for enzymes involved in iron- and nitrogen-metabolism, *Syn-hhoA* with genes coding for enzymes involved in response to heat shock and high light stress, cellular responses (chaperones, proteases) and transcription, while *Syn-hhoB* displays a similar expression pattern with genes encoding enzymes involved in carbohydrate metabolism and periplasmic processes, most probably peptidoglycan and S-layer biosynthesis (Miranda et al., 2013).

### Regulation of Protein Level

Investigations on the whole proteome can be performed using 2D DIGE (Difference in Gel Electrophoresis), in which labeling with different fluorescent dyes allows detection of differences in protein expression between a control and a mutant strain, or between standard and stress conditions. Investigations on the proteome of *Synechocystis* 6803 grown under standard conditions or after exposure to stress have revealed proteins, which are part of a general stress response, as well as proteins specific to a certain stress condition. Expression of the SynDeg proteases of *Synechocystis* 6803 was affected only by stresses having a direct impact on the membrane system, such as heat shock (Slabas et al., 2006; Suzuki et al., 2006) and salt stress (Fulda et al., 2006; Huang et al., 2006). It is interesting to note that pH stress, either acidic or basic pH, has a remarkable effect on the periplasmic proteome, but seems not to affect general stress proteins such as proteases and chaperones, including SynDeg proteases (Kurian et al., 2006; Zhang et al., 2009). This is a puzzling piece of information, since SynHhoA has been localized in the periplasm (Fulda et al., 2000) and its activity is increased at high pH, especially in the presence of divalent cations (Huesgen et al., 2011). This result correlates well with the expression study of Ohta et al. (2005), where none of the *Syn-deg* genes was found to be significantly affected by acid stress.

Gene expression and proteomics data are consistently overlapping: heat shock, salt stress, and high light stress affect the level of mRNAs and their corresponding SynDeg proteins. A more focused approach, testing the presence and the induction of SynDeg proteases using antibodies (Lam et al., 2015) has revealed that, while SynHhoA and SynHhoB are present in equimolar ratios under normal growth conditions, SynHtrA is barely detectable. SynHtrA is strongly up-regulated under high light and heat stress. In agreement with their supposed overlapping functions, upon high light stress SynHhoA accumulated significantly in mutants depleted of either SynHhoB (Δ*hhoB*) or SynHtrA (Δ*htrA*) compared to WT (Lam et al., 2015). Under high light and heat stress SynHtrA showed the highest up-regulation in Δ*hhoB*, however, the amount of SynHhoB decreased in a Δ*htrA* mutant exposed to heat stress. This indicates that their function is not complementary, but rather inversely regulated (Lam et al., 2015). *Synechocystis* 6803 mutant strains lacking any of the three SynDeg proteases compensate for the missing one by up-regulating other serine endopeptidases, such as Clp and Lon proteases (Lam et al., 2015). Using different strategies for the fractionation of cell compartments the SynDeg proteases have been detected in thylakoid membrane (SynHhoA and SynHtrA; Roberts et al., 2012), plasma membrane (SynHhoA, SynHhoB, and SynHtrA; Huang et al., 2006; Roberts et al., 2012), in the outer membrane (SynHtrA; Huang et al., 2004) and in the periplasmic space (SynHhoA; Fulda et al., 2000). The Deg proteases therefore might be active in different metabolons, however, they also might be located within specific membrane patches connecting plasma membrane and thylakoid membrane (thylakoid centers, Nickelsen and Rengstl, 2013). Probing the secretome of *Synechocystis* 6803 with SynDeg specific antibodies it was possible to show that all three proteases are secreted to the exterior of the cells, the secretion being enhanced with culture age (Cheregi et al., 2015), even though none of the SynDeg proteins contains targeting sequences for export.

## Substrate Identification

Substrate preference can be analyzed by the resulting cleavage sites of a protein library. EcDegP and EcDegQ from *E. coli* were found to prefer valine or isoleucine, β-branching residues, at P1 of their cleavage sites (Kolmar et al., 1996) or even threonine and isoleucine (Krojer et al., 2008). Further factors like disulfide-bridges might determine the specificity due to accessibility (Kolmar et al., 1996). Recent studies in *Streptococcus pneumoniae* showed higher frequencies of non-polar residues at the P1 site (Cassone et al., 2012). Recombinant SynHhoA from *Synechocystis* 6803 cleaved PsbO from spinach on two sites: between valine (R1) and lysine (R1′) and between methionine on R1 and threonine on R1′ (Roberts et al., 2012). N-terminal combined fractional diagonal chromatography (COFRADIC) was used to identify the preferred cleavage sites of the three *Synechocystis* 6803 proteases (Lam et al., 2015). Analysis of the neo N-termini produced by the single recombinant peptidases resulted in about 3000 cleavage sites for SynHhoA or SynHtrA, while SynHhoB only produced circa 700 cleavage sites. Statistical analysis of neo N-termini and their proteins showed that all three peptidases prefer to cleave sites with hydrophobic residues. The cleavage sites varied by one to two amino acids within one target showing a low cleavage site specificity of *Synechocystis* 6803 SynDeg peptidases. Highest probability for a cleavage of all three SynDeg proteases was between valine or alanine at P1, and alanine or serine at P1′. Hydrophobic residues were also preferred at positions P2–P4, while glycine and negatively charged amino acids at P1 and arginine at R1′ were unfavored. While analyses of cleavage sites specificities give insights to substrate preference, real substrates cannot be predicted by this information, several thousands of proteins would fit the profile. Furthermore, compartmentation, proximity of substrate and peptidase, activity state of peptidase, and folding state of a protein determine its fate as a possible substrate.

Identifying a protease substrate is fundamental for understanding its biological role. Using proteomics, two types of strategies are employed: gel-based strategies that rely on visual detection of accumulating protein spots in a protease-depleted mutant background (Ünlü et al., 1997; Miranda et al., 2013; Cheregi et al., 2015; Lam et al., 2015) and gel-free strategies that rely on the identification of cleavage products from complex mixtures of proteins that are digested in a solution (Gevaert and Vandekerckhove, 2004; Lam et al., 2015). The 2D DIGE method has been used to identify protein substrates both in single SynDeg deletion mutants (Lam et al., 2015) and in a triple SynDeg deletion mutant (Miranda et al., 2013; Cheregi et al., 2015). Interpretation of the results is complicated because proteomic changes can be caused by secondary effects of the missing protease. Proteins therefore can accumulate in the deletion mutant as possible substrates or they could diminish/disappear due to activation of other proteases in an *in vivo* system. Applying the 2D DIGE method to single and triple *Syn-deg* mutants, several proteins were detected to be possible targets of the proteases. The following proteins were observed to accumulate: a GTP cyclohydrolase (Miranda et al., 2013; Cheregi et al., 2015), the pyruvate kinase 2 (Pyk2), two phosphate ABC transporters (SphX and PstB1’), a serine hydroxymethyltransferase (GlyA) and a guanylate (GMP) synthetase (GuaA; Lam et al., 2015) (**Table 1**). Proteins involved in twitching and motility (PilO, PilA) are down-regulated both in single and triple deletion mutants. The triple *Syn-deg* mutant showed defects in its cell wall, consistent with a decreased amount of proteins (RfbD, Slr1534) involved in cell structure (Cheregi et al., 2015). Increased amounts of neo N-termini for type 1 secretion mechanism components and elements for peptidoglycan and outer membrane lipopolysaccharide biosynthesis were also found in the Δ*hhoB* single mutant using COFRADIC (Lam et al., 2015). More in-depth experiments are needed to confirm these hypothetical substrates, however, for one protein there is experimental evidence being a physiological target of *Synechocystis* 6803 SynDegs (Miranda et al., 2013; Lam et al., 2015): the extrinsic PsbO protein of PSII, which is degraded by a recombinant SynDeg protease after reduction by thioredoxin (Roberts et al., 2012).

**Table 1:** Differentially regulated proteins identified by gel based assay (DIGE; Cheregi et al., 2015; Lam et al., 2015) and gel free assay (COFRADIC; Lam et al., 2015) comparing WT *Synechocystis* 6803 with the single mutants Δ*hhoA*, Δ*hhoB*, and Δ*htrA* or the triple Deg mutant (Δ*deg*), grown at normal conditions.

Method	ΔHhoA		ΔHhoB		ΔHtrA		Δdeg	
				
	Up	down	Up	Down	Up	Down	Up	Down
Gel based assay (DIGE)	Pyk2 Slr0105	UtrA Gap2 Sll1358 LexA Sll1306	Pyk2 SphX PstP1 **GlyA**	**PstS1** UtrA LexA PilA1 **PilO**	Pyk2 **GlyA** AhcY GuA ClpC Slr0105	UrtA Sll1358 LexA Sll1306 **PilO**	NdhI FolE Sll1306 PrsA Gst1 Sll1530	RfbD GlpX Slr1534
Gel-free Assay (COFRADIC)	SucD CpcB Slr1410		Sll0180 **PstS1 PsbO**	**Sll0141** HlyD PilJ FutA2 LpxA HlyA FrpC Gua Pds **FabF**	PilQ Pbp8 **PsbO** CcmM Sll0274	**Sll0141** MrgA **FabF**	-	-


*Underlined are proteins found to be differentially regulated in at least three mutant strains; proteins differentially regulated in at least two mutant strains are shown bold. Only those proteins were considered that were at least two times up- or down-regulated. In the COFRADIC assay, only proteins with more than 100 N-termini were included.*

Secondary effects of protease deletion led to down-regulation of many proteins, with functions in energy metabolism and carbon fixation, photosynthesis, amino acid biosynthesis, stress response and protein quality control (Miranda et al., 2013; Cheregi et al., 2015; Lam et al., 2015). The enzymes Gap2 (Lam et al., 2015), phosphoglycerate kinase, enolase and glpX (Miranda et al., 2013; Cheregi et al., 2015) of the Calvin–Bensson–Basham are down-regulated and negatively affect the production of NADPH. Also, subunits of the ATP synthase (AtpA, AtpD) are down-regulated. Furthermore in the absence of one SynDeg protease other protein quality control factors were differentially regulated (Lam et al., 2015).

Absence of a single SynDeg protease caused opposite effects on the accumulation of some proteins compared to the triple *Syn-deg*-deletion mutant. Pyruvate kinase (Pyk2) and fructose biphosphate aldolase (FbaII) are upregulated in single mutants, while FbaII has been found to be down-regulated in the triple *Syn-deg* mutant, both under normal growth and under stress conditions (Miranda et al., 2013; Cheregi et al., 2015; Lam et al., 2015) pointing to intricate regulatory mechanisms that sense the absence of one or more SynDeg proteases. Sll1306, a putative chitooligosaccharide deacetylase, and ArgC, an enzyme involved in amino acid biosynthesis, are other proteins which are differentially regulated in the single and the triple *Syn-deg* mutant: down-regulated in all single mutants (Lam et al., 2015) and accumulated in the triple mutant (Miranda et al., 2013; Cheregi et al., 2015), while GlyA, a serine hydroxymethyltransferase, with a primary role in folate metabolism, is accumulating in all single *Syn-deg* mutants and is down-regulated in the triple *Syn-deg* mutant.

To identify possible substrates of the *Synechocystis* 6803 SynDeg proteases, a gel-free approach, N-terminal COFRADIC, was performed on *in vivo* and *in vitro* whole cells proteome (Lam et al., 2015). The *in vivo* screening is based on comparing the labeled N-termini of each single *Syn-deg* mutant and WT, while in the *in vitro* approach the N-termini isolated from WT whole cells extracts were compared with and without the addition of one of the recombinant SynDeg proteases (Lam et al., 2015).

Comparing the N-terminal proteome of single *Syn-deg* deletion mutants to wild-type neo N-termini putative substrates should be detectable in wild-type, but not in the mutants. Of the eleven proteins with neo N-termini only in wild-type three proteins also were detected in an *in vitro* COFRADIC approach and therefore might be native substrates of the *Synechocystis* 6803 SynDeg peptidases (Lam et al., 2015): RbcS for SynHhoA, PsbO for SynHhoB as well as for SynHtrA, and Pbp8 for SynHtrA. Comparing the proteomes of single mutants with wild type, deletion of SynHhoA had impact on CO_2_ fixation, while the proteomes of Δ*hhoB* and Δ*htrA* pointed to changes in the photosystems, accumulating PsbO, PsaC, and PsaD (Lam et al., 2015). The SynHhoB single mutant seems to be impaired in its ability to take up phosphate as its PstS1 levels are strongly reduced resulting in defective phosphate sensing (Pitt et al., 2010; Lam et al., 2015). Beside the impact of SynHtrA on photosynthetic proteins its deletion also affects a member of a penicillin-binding protein Pbp8 (Lam et al., 2015), which has been shown to be important in the final step of cell division together with Pbp5 (Marbouty et al., 2009).

## Psii Degradation

Photosystem II is vulnerable to various abiotic stresses such as light or heat. Most likely triggered by reactive oxygen species the most critical damage occurs in the reaction center protein D1 (PsbA). In higher plants the acceptor-side photoinhibition of PSII induces cleavage of the DE-loop of D1, producing a 23- and a 9 kDa fragment (Aro et al., 1993). However, D1 damage occurs even under low light intensity (Tyystjärvi and Aro, 1996). Weak illumination of PSII is enough to produce cationic radicals at the donor side. Furthermore iron–sulfur centers of cytochrome (Jung and Kim, 1990) or more recently the disruption of the Mn-cluster were suggested to contribute to photoinhibition (Hakala et al., 2005; Ohnishi et al., 2005). Two protease families, FtsH and Deg proteases, contribute to the degradation of photo-damaged D1 in plants. The membrane-bound ATP-dependent metallo-proteases FtsH1 and FtsH5 (type A) and FtsH2 and FtsH8 (type B; Zaltsman et al., 2005) form a hexameric complex to degrade their substrate progressively from the terminal end (Lindahl et al., 1996).

Besides FtsH, Deg1, Deg5, Deg7, and Deg8 are major players in the catabolism of Photosystem II in plants. Cooperative D1 degradation by Deg and FtsH has been observed under photoinhibitory conditions (Kato et al., 2012). AtDeg1 is the best characterized HtrA-family member. The protease activity of AtDeg1 leads to the specific cleavage of an exposed loop of photodamaged D1 by the concerted action of other thylakoid proteases (Kapri-Pardes et al., 2007), for reviews, see (Edelman and Mattoo, 2008; Huesgen et al., 2009). D1 fragmentation generated by AtDeg1 was found to be enhanced in mutants lacking FtsH2 in *Arabidopsis* (Kato et al., 2015) and *Chlamydomonas* (Malnoë et al., 2014). Interestingly, AtDeg1 protease is selectively activated under light-stress conditions, during which acidification of the thylakoid lumen occurs and photosynthetic proteins are damaged (Ströher and Dietz, 2008). The importance of AtDeg1 in PSII repair was supported by the finding that this protein also acts as chaperone, assisting in the assembly and biogenesis of PSII by interacting with the PSII reaction center protein D2 (Sun et al., 2010). AtDeg5 and AtDeg8 were shown to form hetero-oligomeric complexes with a 1:1 stoichiometry (Sun et al., 2007). *At-deg5*, *At-deg8*, and even stronger *At-deg5/At-deg8* knock-out mutants of *Arabidopsis* displayed growth inhibition under high light conditions. Pulse-chase experiments visualized the D1 turnover to be impaired in these mutants. AtDeg5 was further shown to be involved in plant development and degradation of PsbF (Cyt *b_559_*) after wounding (Lucínski et al., 2011). Even recombinant AtDeg7 was able to degrade D1, D2, CP43, and CP47 after high light pre-treatment of *Arabidopsis* thylakoids. Analysis of *At-deg7* knock-out plants revealed that they were impaired in growth compared to wild-type plants. This effect was even more pronounced in *At-deg7/At-deg5* and *At-deg7/At-deg8*, but not in *At-deg7/At-deg2* mutants (Sun et al., 2010).

Despite indirect evidence for an involvement of SynDeg proteases in D1 degradation in *Synechocystis* 6803 (Silva et al., 2002; Kanervo et al., 2003), the D1 degradation mechanism seems to differ in plants and cyanobacteria (Nixon et al., 2005; Komenda et al., 2007; Yamamoto et al., 2008). Instead, SynHhoA might be involved in the degradation of D1 protein cross-links (Funk and Adamska, unpublished). Damaged D1 protein aggregates with nearby polypeptides, such as D2, Cyt *b_559_*, and/or CP43 (Lupínková and Komenda, 2004; Komayama et al., 2007) for review, see (Yamamoto et al., 2008). Binding of the extrinsic PsbO protein to Photosystem II has shown to play a regulatory role in directing the damaged D1 protein to degradation or aggregation (Yamamoto et al., 2008). Earlier studies have shown that aggregates of the D1 protein are digested by a stromal serine-type protease (Ishikawa et al., 1999; Ferjani et al., 2001). In pull-down assays all three SynDeg proteases were co-isolated with Photosystem II in sub-stoichiometric amounts (Roberts et al., 2012). Alternatively, the plasma membrane of cyanobacteria may also be a site for D1 degradation and PSII repair (Huang et al., 2002a).

Proteomic comparisons of wild-type *Synechocystis* 6803 cells with single or multiple SynDeg deletion mutants grown under normal (Cheregi et al., 2015; Lam et al., 2015) or stress (Miranda et al., 2013) conditions did not point to an involvement of the SynDeg proteases in D1 degradation, however, the hydrophobic D1 protein might have escaped detection. Instead, biochemical studies showed PsbO to be a substrate of the combined action of SynHhoB/SynHtrA (Lam et al., 2015, see previous paragraph) or under reduced conditions of SynHhoA (Roberts et al., 2012). Even AtDeg1 from *Arabidopsis* was able to degrade this thylakoid lumen protein *in vitro* (Chassin et al., 2002) and *in vivo* (Li et al., 2010). The extrinsic PsbO, PsbP, and PsbQ proteins as well as Mn are released from their binding sites by heat stress (Yamamoto and Nishimura, 1983). Even though PsbO is a highly stable protein (Hashimoto et al., 1996), it is oxidatively damaged under light stress (Henmi et al., 2004).

SynHhoA and SynHtrA Deg proteases have further been shown to be able to degrade *in vitro* the cyanobacterial phycobilisome (Diao et al., 2011) as well as plant light harvesting antenna proteins. AtDeg1 stimulated cleavage of the minor light-harvesting proteins CP26 (Lhcb5) and CP29 (Lhcb4) as well as PsbS (Zienkiewicz et al., 2012). Degradation of CP24 (Lhcb6) was impaired in *Arabidopsis At-deg2* knock-out mutants under short-term stress (salt, wounding, high temperature, and high irradiance; Lucínski et al., 2011).

## Cyanobacterial Deg Proteases Have Impact on the Cell Surface and Might Be Involved in Secretion

The roles of Deg proteases in quality control in the periplasm and at the cell surface are well documented for *E. coli* and many strains of pathogenic bacteria. Also in cyanobacteria, their emerging functions are related to the modification of the outer cell layers (Barker et al., 2006; Cheregi et al., 2015; Lam et al., 2015) and even to the modification of secreted compounds (Cheregi et al., 2015). The triple *Syn-deg*-deletion mutant (Δ*deg*) is affected in phototaxis, probably due to disturbances in pilus biogenesis or in the phototaxis sensory pathways (Barker et al., 2006). Our recent investigation has shown that the intactness of the outermost cell layer, the S-layer, is compromised in this mutant. This hydrophilic layer apparently is absent in Δdeg, consistent with the observed down-regulation of the enzyme GDP-D mannose dehydratase, which affects the synthesis of fucose, mannose and talose (Mohamed et al., 2005; Cheregi et al., 2015). These sugars are components of the glycoproteins of the S-layer and they are also components of secreted exopolysaccharides. Additional evidence supporting the role of SynDeg proteases in the maintenance of extracytoplasmatic properties was obtained in proteomics experiments, demonstrating many proteins related to motility (PilA1, PilJ, PilN, PilO, PilT, and PilQ; Miranda et al., 2013; Cheregi et al., 2015; Lam et al., 2015) and outer cell layer composition (HlyA, Pbp8, Sll1306, Slr1534; Miranda et al., 2013; Cheregi et al., 2015; Lam et al., 2015) to be affected by the deletion of one or all *Syn-deg* genes. As mentioned above, Pbp8, a probable D-alanyl-D-alanine carboxypeptidase with a role in peptidoglycan biosynthesis, is a potential physiological substrate of HtrA (Lam et al., 2015). Two of the most abundant secreted proteins in *Synechocystis* 6803, the hemolysin HlyA and an iron (III) transporter FutA2 (Gao et al., 2014) are much stronger degraded in Δ*hhoB* compared to wild type. In general an increased degradation of secreted proteins was observed in the absence of SynDeg proteases (Cheregi et al., 2015; Lam et al., 2015). Inefficient secretion might induce the higher degradation rate by other proteases in the mutant. Similar to pathogenic strains (Biswas and Biswas, 2005), in *Synechocystis* 6803 SynHhoB (and even SynHtrA and/or SynHhoA) might work as chaperones in the periplasm protecting the cell from aggregated proteins (Lam et al., 2015).

Few glycolytic enzymes with altered expression in the single or triple *Syn-deg* deletion mutants are known to be secreted both, in cyanobacteria (Hahn et al., 2014; Vilhauer et al., 2014; Cheregi et al., 2015) and pathogenic bacteria (reviewed by Henderson and Martin, 2011). Despite lacking any identifiable secretion signal these glycolytic enzymes (enolase, phosphoglycerate kinase, fructose-biphosphate aldolase) have been found to be associated with the surface of bacteria where they exhibit non-glycolytic functions (Henderson and Martin, 2011; Mani et al., 2015). The ability of a protein to have more than one biological function has been termed “moonlighting.” Moonlighting activity depends on the presence of more than one biochemical active site on a protein and may occur only at a cellular location different from the one where the protein is commonly found (Henderson and Martin, 2011). Besides the described impact on metabolism, deletion of one or all SynDeg protease(s) could affect moonlighting functions of other enzymes, e.g., these glycolytic enzymes.

Our hypothesis that the *Synechocystis* 6803 SynDeg proteases function in remodeling the cell exterior is emphasized by the finding that all three proteases are secreted into the media from cells growing in stationary phase. Secretion of SynDeg proteases is part of the virulence mechanism in many pathogenic, Gram-negative bacteria (Biswas and Biswas, 2005; Hoy et al., 2012). However, proteolytic activity in the extracellular media is not an exclusive feature of pathogenic strains: production of extracellular material and acquisition of nutrients require remodeling based on proteolysis (Havarstein et al., 1995).

## Chaperone Activity

Intracellular protein concentrations are high leading easily to protein aggregation. Molecular chaperones primarily work as cellular defense factors preventing and averting mutation- and stress-induced accumulation of misfolded and aggregated proteins. These quality control factors distinguish between substrates that can be refolded and damaged proteins that have to be degraded. Deg proteases are of particular interest as many family members combine the dual activities of chaperones and proteases. While other enzymes with combined protease/chaperone function contain special subunits or domains for their chaperone activity (e.g., ClpAP of the Clp protease or the AAA-domain of Lon or FtsH proteases), Deg proteases are ATP-independent and combine their antagonistic functions in the same protein. Their PDZ domains act as gatekeepers (see paragraph above) and in this way permit a direct coupling of substrate-binding and subsequent translocation into the inner chamber, which seems to be highly important for the chaperone activity.

In *E. coli* this dual function as chaperone and protease has been shown to be tightly regulated (Spiess et al., 1999; Clausen et al., 2002). *In vitro* studies demonstrated a temperature-switch between the two functions (Spiess et al., 1999); EcDegP acts as a chaperone at temperatures below 28°C, whereas it has protease activity at higher temperatures. The switch in activity should mainly depend on the interplay of loops LA and L2. Especially, an extended L2 loop seems to be required for protease inhibition (Clausen et al., 2002). The physiological relevance of EcDegP acting as chaperone has been supported *in vivo* (Clausen et al., 2002). Tight control of chaperone and protease function is of pivotal importance in preventing deleterious HtrA protease activity (for review, see Hansen and Hilgenfeld, 2013). The chaperone/protease regulation therefore is archived not only by temperature: Since the expression of *Ec-deg*P is regulated by σ^E^, which in turn is activated by EcDegS, the concerted action of EcDegS and EcDegP permits recognition and relief of periplasmic protein-folding stress. Under non-stressed conditions, EcDegP encapsulates folded monomers of outer-membrane proteins and participates in guiding them through the periplasm protecting them from proteolytic degradation during their passage through the periplasm (Sawa et al., 2011). It further promotes folding of proteins (Spiess et al., 1999) and prevents aggregation of misfolded ones (Shen et al., 2009).

Even EcDegQ is combining chaperone and protease activities (Wrase et al., 2011). Single particle cryo-EM data visualized up to six substrates densely bound inside a EcDegQ 12-mer, their PDZ-domains located adjacent to substrate density (Malet et al., 2012). The EcDegQ cage assembly is triggered by substrate binding. Chaperone activity has further been shown for HtrA of yeast (Ynm3 or Nma111; Padmanabhan et al., 2009), of *Chlamydia trachomatis* (Huston et al., 2011) and plant AtDeg1 (Sun et al., 2010), while human HtrA2/Omi has been shown to display protease activity at room temperature (Savopoulos et al., 2000).

In *Synechocystis* 6803 a temperature switch between chaperone- and protease function has not been observed (Huesgen et al., 2011). However, recombinant SynHhoB, which has very low proteolytical activity (Huesgen et al., 2011; Lam et al., 2015), was able to assist folding of denatured MalS at normal temperature (Lam et al., 2015). Absence of HhoB in a *Synechocystis* 6803 mutant leads to different expression of several periplasmic proteins, especially transporters or signaling components (Lam et al., 2015). Chaperones are known to be important for protein secretion (Gottesman, 1996). The location of SynHhoB in the plasma membrane (Roberts et al., 2012) and the co-expression of its corresponding gene with a wide range of periplasmic proteins (Miranda et al., 2013) support its function within secretion. SynHhoB itself is secreted in cultures growing in the stationary phase (Cheregi et al., 2015). Notably, many proteins differentially regulated in a *Syn-hhoB-*deletion mutant are predicted to enter the periplasm in an unfolded state through the general secretory (SEC) pathway (Lam et al., 2015). Interesting is the fact that during stress *in vivo* SynHhoB activity seems to be tightly connected to SynHtrA activity. The down-regulation of the chaperone SynHhoB accompanied by an up-regulation of the protease SynHtrA (Lam et al., 2015) during stress make it tempting to speculate about a hetero-oligomeric unit of SynHhoB/SynHtrA, which switches function from chaperone to protease similar to the temperature switch observed for the homo-oligomeric EcDegP in *E. coli* or HtrA in *Thermotoga maritima* (Spiess et al., 1999; Kim et al., 2003). A similar thermal shift even was observed between two forms of SynHhoA with slightly different molecular mass (Lam et al., 2015); the amount of SynHhoA with higher molecular mass increased, whereas the lower molecular mass form decreased during heat stress. Further studies on the chaperone functions of the three cyanobacterial SynDeg proteases are necessary to gain more insight.

## Future Perspectives

Deg proteases are important ATP-independent quality-control factors. Members from the Deg family in *H. sapiens* were associated with different cancer types, Parkinson’s and Alzheimer’s diseases. Many pathogenic bacteria strains lacking the Deg/HtrA function lose virulence or their virulence is decreased due to an increased vulnerability to stresses or to a decrease in secretion of virulence factors. In some prokaryotes, including *Synechocystis* 6803, Deg proteases are secreted outside the cell. Thus, the HtrA proteases of bacterial pathogens are relevant targets for new therapeutic approaches as they promote the pathogen’s invasiveness. Their ability to control the cell surface in *Synechocystis* 6803 might be usefull for the production of green energy and higher value products in cyanobacteria. In photosynthetic organisms, the Deg proteases have been shown to be important for quality control of Photosystem II and thereby in biomass production.

To fully understand the function of cyanobacterial Deg proteases it is important to unambiguously identify their subcellular localization. Visualizing the Deg proteases *in vivo* by GFP-tagging unfortunately was not successful in our hands (Lam et al., unpublished results). Other approaches, e.g., gold immuno labeling, therefore will be necessary to localize SynHhoA, SynHhoB, and SynHtrA. An important property of *E. coli* Deg proteases is their ability to discriminate between polypeptides that still can be refolded or need to be degraded. This chaperone/proteases switch might even be present in *Synechocystis* 6803, performed by a SynHhoB/SynHtrA heteromer. More work on the chaperone activity of the SynDeg proteases is necessary to understand this phenomenon. For this even structural details of cyanobacterial homo- or hetero-oligomers will be needed. Finally, the search for additional native substrates and regulators will contribute to our understanding of this fascinating protease family.

## Methods

### Alignment

All analyzed sequences derived from CyanoBase (Nakamura et al., 1998). Mature sequences from *Synechocystis* sp. PCC 6803 SynDeg proteases were obtained, the predicted signal peptides according to Pred-Tat (Bagos et al., 2010) was excluded. Sequences of mature SynHtrA, SynHhoB, and SynHhoA then were blasted against the Cyanobase database using blastp 2.2.26 (Altschul et al., 1997; Altschul et al., 2005). Candidate sequences with high similarity to the Deg proteases of *Synechocystis* 6803 were screened for the Interproscan domain PR00834 indicating a member of the MEROPS subfamily S1C^2^ (Rawlings et al., 2012). The focus was set on species designated as cyanobacteria, gene products lacking PDZ domains were included. The resulting protein list was analyzed via Pred-Tat (Bagos et al., 2010) and predicted signal peptides were excluded. Mature protein sequences were aligned via MUSCLE (Edgar, 2004), gap open -2.9, gap extend 0, hydrophobicity multiplier 1.2, clustering method UPGMP, lambda 24.

### Molecular Phylogenetic Analysis by Maximum Likelihood Method

The evolutionary relationship was inferred by using the Maximum Likelihood method with software PhyML 3.1 (Guindon et al., 2010) based on the Le_Gascuel_2008 model (Le and Gascuel, 2008). The bootstrap consensus tree was inferred from 1000 replicates (Felsenstein, 1985). The initial tree for the heuristic search was obtained by applying the Bio-Neighbor-Joining method. A discrete Gamma distribution was used to model evolutionary rate differences among sites [four categories (gamma shape parameter 0.729)], the analysis involved 124 amino acid sequences. Tree visualization was done in MEGA6 (Tamura et al., 2013). Full taxon names are described in **Supplementary Table S1**. Branches with frequencies equal or higher than 70% were labeled with a black circle.

### Homology Modeling

The sequence of mature SynHhoB of *Synechocystis sp.* PCC 6803 or sequences of its domains were analyzed via the Phyre2 web portal for protein modeling, prediction and analysis (Kelley et al., 2015). For modeling the intense mode of Phyre2 was chosen. The *in silico* SynHhoB model formed after the DegQ trimer was chosen (RCSB designator 3STJ; Sawa et al., 2011) to visualize the domain elements. The same model was taken as a template to overlay the SynHhoB models formed after Deg1 (3qo6), Deg2 (4fln), and Deg8 (4ic6). Resulting superpositions were visualized with software Chimera 1.10.2 (Pettersen et al., 2004).

## Author Contributions

All authors listed, have made substantial, direct and intellectual contribution to the work, and approved it for publication.

## Conflict of Interest Statement

The authors declare that the research was conducted in the absence of any commercial or financial relationships that could be construed as a potential conflict of interest.

## Footnotes

1 http://www.ebi.ac.uk/interpro/

2 http://www.ebi.ac.uk/interpro/interproscan.html

## Supplementary Material

**TABLE S1 | Nomenclature information of cyanobacterial strains used in the phylogenetic tree in **Figure 1**.**

**TABLE S2 | Summary of Deg genes expression in microarray and RNA sequencing experiments extracted from the Cyanoexpress (Hernandez-Prieto and Futschik, 2012).**
